# Role of pinch in Argon impurity transport in ohmic discharges of Aditya-U Tokamak

**DOI:** 10.1038/s41598-023-42746-2

**Published:** 2023-09-26

**Authors:** K. Shah, J. Ghosh, S. Patel, M. B. Chowdhuri, K. A. Jadeja, G. Shukla, T. Macwan, A. Kumar, S. Dolui, K. Singh, R. L. Tanna, K. M. Patel, R. Dey, R. Manchanda, N. Ramaiya, R. Kumar, S. Aich, N. Yadava, S. Purohit, M. K. Gupta, U. C. Nagora, S. K. Pathak, P. K. Atrey, K. B. K. Mayya

**Affiliations:** 1 Department of Physics, Pandit Deendayal Energy University, Raisan, Gandhinagar, 382 007 India; 2 Institute for Plasma Research, Bhat, Gandhinagar, 382 428 India; 3 Homi Bhabha National Institute, Training School Complex, Anushaktinagar, Mumbai, 400 094 India; 4 Department of Nano Science and Advanced Materials, Saurashtra University, Rajkot, 360 005 India; 5 ITER-India, Institute for Plasma Research, Koteshwar, Ahmedabad, 380 005 India; 6 University of California, Los Angeles, CA 90095 USA; 7 Institute of Science, Nirma University, Ahmedabad, 382 481 India

**Keywords:** Magnetically confined plasmas, Physics

## Abstract

We present experimental results of the trace argon impurity puffing in the ohmic plasmas of Aditya-U tokamak performed to study the argon transport behaviour. Argon line emissions in visible and Vacuum Ultra Violet (VUV) spectral ranges arising from the plasma edge and core respectively are measured simultaneously. During the experiments, space resolved brightness profile of Ar^1+^ line emissions at 472.69 nm (3p^4^4s ^2^P_3/2_–3p^4^4p ^2^D_3/2_), 473.59 nm (3p^4^4s ^4^P_5/2_–3p^4^4p ^4^P_3/2_), 476.49 nm (3p^4^4s ^2^P_1/2_–3p^4^4p ^2^P_3/2_), 480.60 nm (3p^4^4s ^4^P_5/2_–3p^4^4p ^4^P_5/2_) are recorded using a high resolution visible spectrometer. Also, a VUV spectrometer has been used to simultaneously observe Ar^13+^ line emission at 18.79 nm (2s^2^2p ^2^P_3/2_–2s2p^2^ ^2^P_3/2_) and Ar^14+^ line emission at 22.11 nm (2s^2^ ^1^S_0_–2s2p ^1^P_1_). The diffusivity and convective velocity of Ar are obtained by comparing the measured radial emissivity profile of Ar^1+^ emission and the line intensity ratio of Ar^13+^ and Ar^14+^ ions, with those simulated using the impurity transport code, STRAHL. Argon diffusivities ~ 12 m^2^/s and ~ 0.3 m^2^/s have been observed in the edge (ρ > 0.85) and core region of the Aditya-U, respectively. The diffusivity values both in the edge and core region are found to be higher than the neo-classical values suggesting that the argon impurity transport is mainly anomalous in the Aditya-U tokamak. Also, an inward pinch of ~ 10 m/s mainly driven by Ware pinch is required to match the measured and simulated data. The measured peaked profile of Ar density suggests impurity accumulation in these discharges.

## Introduction

In addition to the fuel ions, tokamak plasmas are inherently comprised of multiple non-fuel ion species originating from plasma-material (vacuum vessel wall and other peripherals surrounding the plasma) interactions^1^ and also from the intentionally seeded non-fuel ion species^2^. The high-Z material walls of the present-day and future fusion devices, such as tungsten etc., lead to introduction of high-Z ion species into the core plasma, which can have adverse effects on the performance and operational capabilities of a tokamak, even leading to plasma disruptions^3^. On the other hand, some ion species of relatively low-Z, such as nitrogen^4,5^, neon^6^, argon^7^ are deliberately injected into the plasma, so-called the ‘impurity-seeding’, to achieve low-H-mode thresholds^7,8^ as well as to minimize and control the heat loads and heating of the peripheral materials through radiative power dissipation at the plasma boundary. Trace impurity seeding is also done for diagnostics purposes^4–11^.

However, both of these, peripheral-material-generated and seeded ion species, colloquially known as ‘impurities’, adversely affect the entire plasma discharge, when they reach to the core of the plasma column and accumulate there. Accumulated impurities radiate the core plasma energy via line radiation and continuum radiation, such as bremsstrahlung and recombination radiation^12^, resulting in the confinement degradation and fuel dilution. One of the crucial challenges to sustain long-pulse high performance operation of tokamaks is to control the impurity concentration and accumulation inside the plasma column as well as their dynamics inside the plasma. Controlling impurities inside the plasma is an urgent and critical issue for ITER^1^ also and therefore, understanding and finding ways to control impurity concentration^2^ inside the plasma core have received much attention in the fusion community in recent times.

On the other hand, impurities such as nitrogen, neon and argon are injected in several tokamaks including JET^7^, TEXTOR-94^13^, TFTR^14^, DIII-D^15^, ASDEX-U^16^, Aditya-U^17^, EAST^18^, KSTAR^9^, MAST^10^, T-10^11^ etc. for radiative divertor cooling and heat-load reduction to the tugnsten targets^19^ as well as to lower the H-mode threshold in presence of metallic walls^7^. Impurity seeding experiments led to additional results, such as the observation of radiative improved (RI) confinement mode^7,14–16,20^. Impurities are also puffed or injected by the laser blow-off technique to study impurity transport inside the plasma column^21^. Interestingly, it has been observed that a particular impurity specie show different dynamics in different discharge regimes, such as, in ohmic (Linear and Saturated ohmic confinement regimes), L-mode and H-mode. The transport and accumulation of a particular impurity also depend on the auxiliary heating schemes and the radial locations of heat injection^22^. Also, different impurities behave differently inside the tokamak plasma even in a similar collisionality regime. For example, neon and argon behave differently in similar discharges of TCV tokamak^23^.

Even though, extensive amount of research work has been carried out to understand the impurity transport phenomenon associated with different impurities, the underlying mechanisms are not yet completely understood and the explanation varies from device to device, depending on the impurity species and also on the operating regime. The experimentally observed impurity transport is found to be often deviating from collisional predictions. Convection and plasma turbulence are invoked as reasons for anomalous impurity transport^9–11,22^. Substantial progress has been made in exploring possible mechanisms for turbulence-driven impurity transport and neoclassical transport including the centrifugal effects for heavy impurity ions^24^.

Argon is one such impurity which is seeded in tokamaks for edge radiative cooling experiments to reduce the heat-loads on divertor plates^2^. The Argon gas-puff is also used specifically for impurity transport studies as well as for diagnostic purposes mainly for measurements of plasma rotation and ion temperature using the X-ray crystal spectrometers^25,26^. However, not much work is specifically dedicated on Ar transport except in few devices^9,11,27–29^. In ohmic plasmas of T-10 tokamak, argon impurity transport has been assessed by measuring the spectral line emissions of Ar^15+^, Ar^16+^ and Ar^17+^ ions. This study revealed that the introduction of an anomalous transport term leads to central impurity removal, consistent with the experimental results^11^. In the ohmic discharges of TEXTOR tokamak, argon puffing experiments are performed and argon transport has been assessed modelling the measured argon spectral line emissions in the VUV and X-ray range using the STRAHL code. The nature of transport has been found to be purely anomalous at various densities ranging from 1.4 ~ 3.5 × 10^19^/m^3^ in these experiments^30^. Argon is also injected in KSTAR tokmak through gas-puffs. X-ray emission measured by multi-channel soft x-ray (SXR) array diagnostic system together with SANCO impurity transport code are used to understand the core argon impurity transport in the L-mode^9^ and H-mode^31^ discharges in absence and presence of the electron cyclotron resonance heating [ECRH]. It has been shown in these experiments that the argon accumulation in the core-plasma can be altered with on-axis ECRH with argon ions reversing their convection direction with ECRH. The charge exchange spectral line emissions from fully stripped argon ions and VUV emissions of Ar^14+^ and Ar^15+^ have been measured and simulated to comprehend the Ar impurity transport in the argon seeded discharges of the JET tokamak^27^. It has been reported that argon impurity accumulation in the core varies significantly in different discharge scenarios such as in discharges with neutral beam injection (NBI) heating, and in combination of Ion Cyclotron Resonance Heating (ICRH) and NBI. In almost all the discharge scenarios, significant anomalous contribution to the diffusivity of argon inside the plasma has been reported. In ASDEX-Upgrade tokamak, hollow radial profiles of argon density in the ECR heated L-mode discharges has been reported by modelling the soft X-ray signals and Ar X-ray lines using the STRAHL impurity transport code^28^. In an attempt to identify different argon spectral line emissions in the visible and VUV range of wavelengths, argon plasmas are produced in the large helical device (LHD) Katai et al^32^.

Argon gas-puffing experiments are carried out to study the behaviour of argon impurity transport in the ohmic discharges of Aditya-U tokamak. Trace amount of Ar is injected by gas-puffing during the plasma current flat-top phase in the purely ohmically heated discharges. Multiple spectroscopic line emissions from different charge states of argon in the visible and vacuum ultraviolet (VUV) range have been measured. The visible and VUV line emissions from various ionization stages of Ar impurity can be used to study its transport in the edge and core regions simultaneously in the small and medium sized tokamaks due to relatively low energy and temperatures. In a typical Aditya-U discharge with Ar gas puff, radial profiles of multiple line emissions of Ar^1+^ in the visible range at 472.69 nm (3p^4^4s ^2^P_3/2_–3p^4^4p ^2^D_3/2_), 473.59 nm (3p^4^4s ^4^P_5/2_–3p^4^4p ^4^P_3/2_), 476.49 nm (3p^4^4s ^2^P_1/2_–3p^4^4p ^2^P_3/2_), 480.60 nm (3p^4^4s ^4^P_5/2_–3p^4^4p ^4^P_5/2_) have been measured. Furthermore, chord-averaged spectral line emissions of B-like Ar (Ar^13+^) and Be-like Ar (Ar^14+^) in the VUV range are also measured. The measured radial profiles of Ar^1+^ line emission and the measured ratio of two VUV spectral emissions of Ar^13+^ at 18.79 nm (2s^2^2p ^2^P_3/2_–2s2p^2^ ^2^P_3/2_) and of Ar^14+^ at 22.11 nm (2s^2^ ^1^S_0_–2s2p ^1^P_1_) are simulated using the STRAHL code^33^ to estimate the diffusivity and convective transport parameters of Ar in the Aditya-U plasma. The simultaneous measurements of Ar^1+^ emission from the edge region provides the boundary condition to the simulation code and hence appropriately estimating the Ar input to the code. It has been found that the argon transport remains anomalous throughout the plasma column in the ohmically heated discharges of Aditya-U tokamak. Also, only diffusivity is not sufficient to match the measurements. Convective velocity is essential to fit the spectra of Ar^1+^ and the ratios of Ar^13+^ and Ar^14+^ emissions. The convection seems to be due to increase in the Ware pinch after the injection of Ar. This convection also seems to be responsible for the observed argon concentration in the core of these discharges leading to peaked radial profile of Argon inside the plasma.

## Method of Argon transport analysis

Estimation of transport coefficients in Aditya-U. The diffusivity and convective velocity of argon impurity ion have been obatined by comparing the measured spectral emissions of impurity ions with the simulated emissions using one-dimensional impurity transport code, STRAHL via an iterative method. The STRAHL code solves the following continuity equation for each charge state Z of the impurity ion^33^:

1 $$\frac{{\partial_{{n_{Z} }} }}{{\partial_{t} }} = \frac{1}{r}\frac{\partial }{{\partial_{r} }}r\left( {D_{Z} \frac{{\partial_{{n_{Z} }} }}{{\partial_{r} }} - v_{Z} n_{z} } \right) + Q_{z}$$

where $n_{Z}$ is the impurity density and the impurity flux of a charge state *Z* is defined by the diffusivity $D_{Z}$ and the convective velocity $v_{Z}$. $Q_{Z}$ represents sources and sinks of impurities and is given by $Q_{Z} = - (n_{e} S_{Z} + n_{e} \alpha_{Z} + n_{H} C_{Z} )n_{Z} + n_{e} S_{Z - 1} n_{Z - 1} + (n_{e} \alpha_{Z + 1} + n_{H} C_{Z + 1} )n_{Z + 1}$. The symbols $C$, $\alpha {\text{and }}S$ represent the reaction rate coefficients for the charge exchange, recombination (radiative and di-electronic) and ionization respectively. The inputs to the STRAHL code are impurity source rate, toroidal magnetic field, initial radial profiles of convective velocity ($v_{Z}$) to diffusivity ($D_{Z}$) ratio ($v_{Z} /D_{z}$), atomic data for ionization and recombination and measured radial profiles of electron temperature and density. In the simulation, transport coefficients, $D_{Z}$ and $v_{Z}$, are presumed to be the same for all charge states. Furthermore, the steady state nature of plasma is considered throughout the simulation time and transport coefficients are considered to be independent of time. As mentioned in the following section the plasma parameters such as electron density and temperature do not vary significantly with time after the argon injection and hence the assumption of steady state of plasma is justified^9–11^.

First, using the radial profiles of electron density and temperature of the plasma, an initial guess of radial profiles of convective velocity ($v$) to the diffusivity ($D$) ratio ($v/D$) and the atomic data for ionization and recombination for argon have been provided to the STRAHL code to compute the ground state density profile for each charge state of argon. After obtaining the ground state densities of all argon charge states, the emissivity, ${\upvarepsilon }_{ij}$, of a specific transition, the transitions which are measured, is obtained using ${\upvarepsilon }_{i,j}^{{{\lambda }}} \left( r \right) = n_{e} \left( r \right)n_{Z,i} \left( r \right){\upvarepsilon }^{exc} \left( r \right)$. Here $n_{Z}$ and $n_{e}$ are the impurity and electron densities, respectively; $\upvarepsilon ^{exc}$ is the photon emissivity coefficient (PEC), which depends on both electron temperature and density and is obtained from the Atomic Data and Analysis Structure (ADAS) database^34^. Following that, the calculated emissivities of the visible and VUV line emissions is matched with the measured emissivities. This process has been iterated by varying the values of ($v/D$) ratio until the simulated emissivity profiles of Ar^1+^ line emission and the intensity ratio of Ar^13+^ and Ar^14+^ line emissions are completely matched with the experimentally measured emissions.

## Aditya-U Tokamak and diagnostics

### Aditya-U tokamak

Aditya-U^35^ is a medium-sized air-core tokamak having stainless steel vacuum vessel with major (R) and minor (a) radii of 0.75 m and 0.25 m, respectively. The maximum toroidal magnetic field, B_T_ = 1.5 T. The parameters of circular ohmic plasmas obtained in limiter configuration with a high-field side toroidal belt limiter in the Aditya-U tokamak are: plasma current ~ 100–250 kA, plasma duration of ~ 100–350 ms with electron density and temperature in the range of 1–3.5 × 10^19^ m^−3^ and 220–500 eV respectively^36^.

### Standard diagnostics

In the reported experiments, the central chord-averaged electron density has been measured using a 100 GHz heterodyne microwave interferometer^37^. For measuring the radial electron density profile, a four-channel homodyne microwave interferometer system, having the viewing chords passing through 0, 7, 14 and 21 cm of plasma minor radius with a temporal resolution of ~ 10 μs has been used^38^. An Abel-like matrix inversion technique is used to get the radial profile of electron density from the chord averaged measurement^39^. The central chord-averaged temperature is measured from SXR emissions detected using AXUV photodiodes using the absorption foil-ratio technique^40^. The Beryllium foils of thickness 10 μm and 25 μm have been used for the temperature estimation. Additionally, integrated SXR emission from every discharge has also been monitored using one surface barrier detector, which mainly views the whole plasma cross-section through a pin-hole. A Berylium foil is placed in front of the surface barrier detector, having a ~ 0.8 keV cutoff (~ 10% transmission).

**Figure 1:**
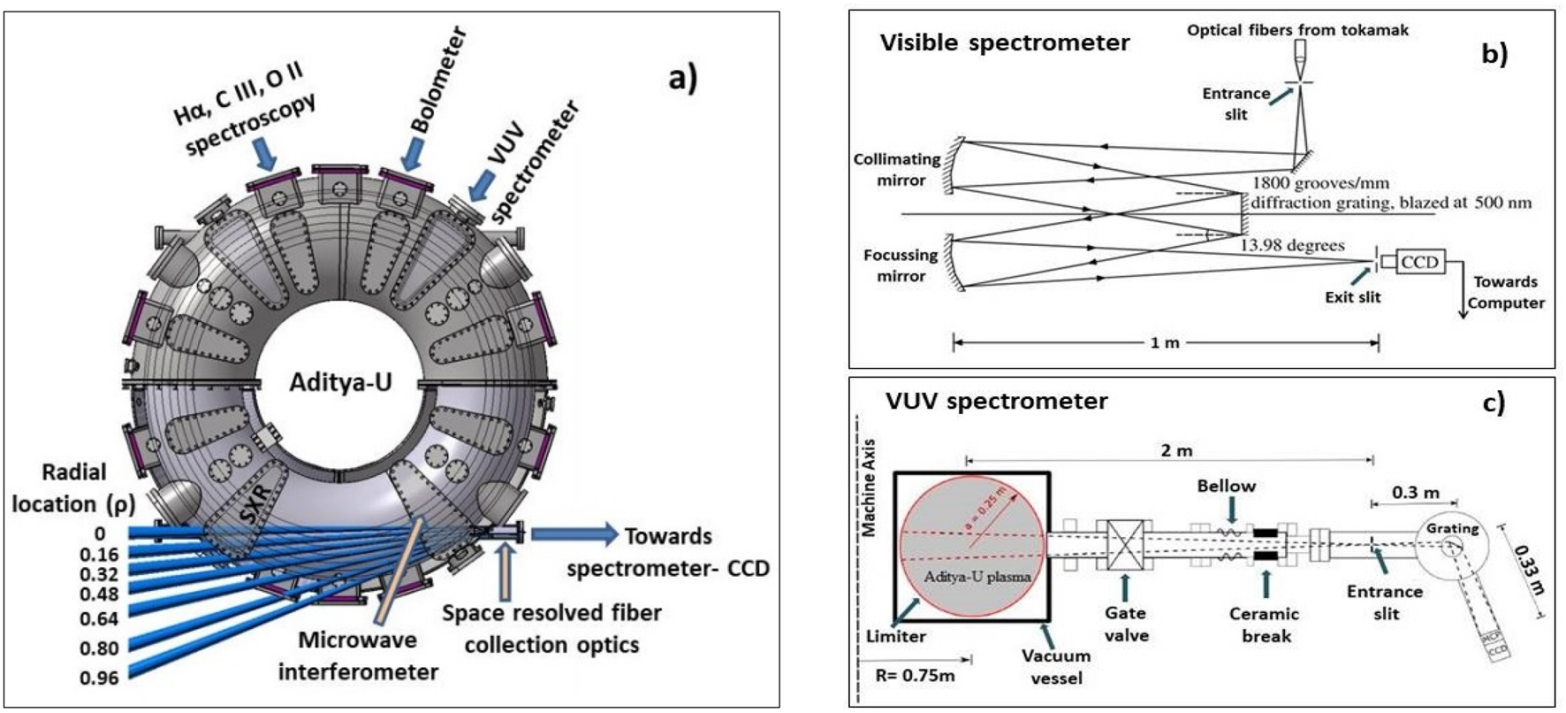
(**a**) Top view of the Aditya-U tokamak showing location of various diagnostics including lines of sight of the space resolved visible spectrometer and VUV spectrometer used for Argon transport study (**b**) Schematic showing space resolved Czerny Turner configuration type visible spectrometer and (**c**) Top view of the VUV spectrometer installed radially on Aditya-U showing various system components.

The H_α_ (656.3 nm) and impurities line emissions such as C^2+^, O^+^ are routinely recorded by the optical setup consisting of lens, optical fiber, interference filter and Photo Multiplier Tube (PMT) detector^41^. The visible continuum is measured by recording the bremsstrahlung emission in the spectral line-free wavelength region of ~ 536 nm^42^. The plasma current is measured by a Rogowski coil positioned inside the vacuum vessel. Similarly, the loop voltage is measured using four flux loops, each containing a single-turn copper wire, placed at four different locations on the vacuum vessel. The plasma stored energy is measured using compensated diamagnetic loops^43^. The edge-plasma diagnostics consist of sets of Langmuir probes placed along the poloidal periphery at different toroidal locations. These probes measure the temporal evolution of the spatial profile of temperature, density and floating potential in the edge and in the Scrape-Off-Layer (SOL) regions. Two poloidal rings containing 16 Mirnov coils at equal intervals are mounted inside the vessel at two toroidal locations to measure the MHD oscillations. All the signals has been acquired and sampled at a frequency of 100 kHz. The radial variation of all quantities are presented with respect to the normalised radius ρ = r/a, where a is minor radius of the plasma column. The locations of various diagnostics over the toroidal periphery of Aditya-U is shown in Fig. 1a.

### Space resolved high resolution visible spectrometer

For the argon transport studies, a recently upgraded high resolution visible spectroscopic diagnostic system has been used to record the space resolved visible spectral lines from singly ionized argon from the Aditya-U plasma with argon injection^17^. The system consists of a 1.0 m and *f*/8.7 Czerny–Turner type spectrometer with a 1800 grooves/mm grating blazed at 518 nm coupled with a charge coupled device (CCD) detector (Model DU 440-BU, Andor) having 2048 × 512 pixels and each pixel size of ~ 13.5 μm. The reciprocal linear dispersion is 0.0061 nm/pixel at 480 nm^44^. The optical schematic of the visible spectrometer is shown in Fig. 1b. For the present study, the width of the entrance slit of the spectrometer is kept at 100 μm. The visible spectrometer is characterised using a Mercury (Hg) calibration lamp. The instrumental width is ~ 0.03 nm at 100 μm slit width. The CCD detector is cooled down to − 40 °C to reduce the thermal noise. With these system parameters, the spectrometer is used to record several Ar^1+^ transitions in the wavelength range between 470 and 482 nm. Absolute intensity calibration of the system has been performed using an integrating sphere to carry out quantitative analysis of line emissions. The intensity calibration has been performed from both inside and outside the tokamak and the transmission of the vacuum window in the wavelength range of interest is taken into account for intensity estimation of Ar^1+^.

To increase the plasma radial coverage, combination of lens and optical fiber, has been placed inside a re–entrant viewport, made up of fused-silica glass, attached to the Aditya-U tokamak’s tangential port. This collection optics provides seven lines of sight (LoSs), having tangential-radius of 0, 4, 8, 12, 16, 20 and 24 cm at the low field side of plasma, covering entire plasma minor radius towards the low-field side with a radial resolution of ~ 2.5 cm as shown in Fig. 1a. The radial locations of the chords are mentioned with respect to geometric centre of the machine. Transported light via optical fibers is coupled to the entrance slit of the spectrometer. The complete details on the upgraded system including in situ measurement of radial locations can be found in^45^.

### High resolution VUV spectrometer

Along with space resolved visible spectroscopic diagnostic, a single chord Vacuum Ultra-Violet (VUV) spectroscopy system (Horiba Jobin–Yvon, Type TGS 330, France) is available in Aditya-U and is routinely operated to record the spectral line emissions from various ionization stages of intrinsic and seeded impurities such as carbon, oxygen, iron, neon, argon in the VUV wavelength range of 10–180 nm. The spectrometer is mounted on the radial port of tokamak at the vertical mid-plane, and is positioned horizontally to view the plasma radially. The schematic of the VUV system is shown in Fig. 1c. The spectrometer has focal length of 0.3 m and is operated under ultra high vacuum (UHV) to observe the VUV line emissions. It consists of three toroidal gratings having groove densities of 290, 450 and 2105 grooves/mm. Entrance slit width can be adjusted between 10 and 250 µm. The gratings used in the spectrometer are Type IV diffraction gratings in which the holographically recorded grooves are curved to place the desired spectral range on a flat focal field with optimal resolution. Furthermore, toroidal mirrors are used to avoid mismatch of curvatures and hence the astigmatism for obtaining a flat focal field following the methodology described in^46^. The dispersed light is detected by a combination of multi-channel plate (MCP) and CCD having 1340 × 255 pixels with pixel size of 20 µm. The system views ~ 7.5 cm along the toroidal direction at the vertical mid-plane of the plasma. During the present experiments, entrance slit width is kept at 30 µm and grating with 2105 grooves/mm has been used for measurements, which has provided a reciprocal linear dispersion of 0.020 nm/pixel. With these VUV system parameters, B and Be-like Argon line emissions between 10 and 30 nm have been recorded. To carry out quantitative analysis of the VUV line emissions, absolute intensity calibration of the VUV spectrometer is performed using a combined conventional branching ratio and collisional-radiative modelling techniques^47^ for the selected spectral transitions of Ar^13+^ and Ar^14+^ having maximum measured intensities in the typical discharges of Aditya-U with argon injection.

## Measurement results

Impurity seeding experiments in the Aditya-U have been carried out in the limiter discharges with hydrogen as the main fuel gas pre-filled at the pressure of ~ 1–2 × 10^–4^ Torr. Argon gas, in appropriate amount, has been injected into the edge plasma region using a pulse gas feed system. The system consists of a programmable pulse generator and a piezo electric valve attached on bottom port of the tokamak. Argon has been injected during the steady state phase of plasma discharge by applying a pre-defined pulse amplitude and duration. The quantity of seeded Ar has been adjusted to record the argon line emissions having good intensities while avoiding the plasma disruption. During typical discharge, Argon particles of ~ 10^16^–10^17^ are injected into the plasma, which is 0.1–1% of the hydrogen gas particles. The amount of injected particles are estimated by measuring the increase in pressure when argon gas-puffs of similar pulse amplitude and duration, as used in the actual experiments, are applied in the vessel having base vacuum of ~ 5–8 × 10^–9^ Torr.

**Figure 2:**
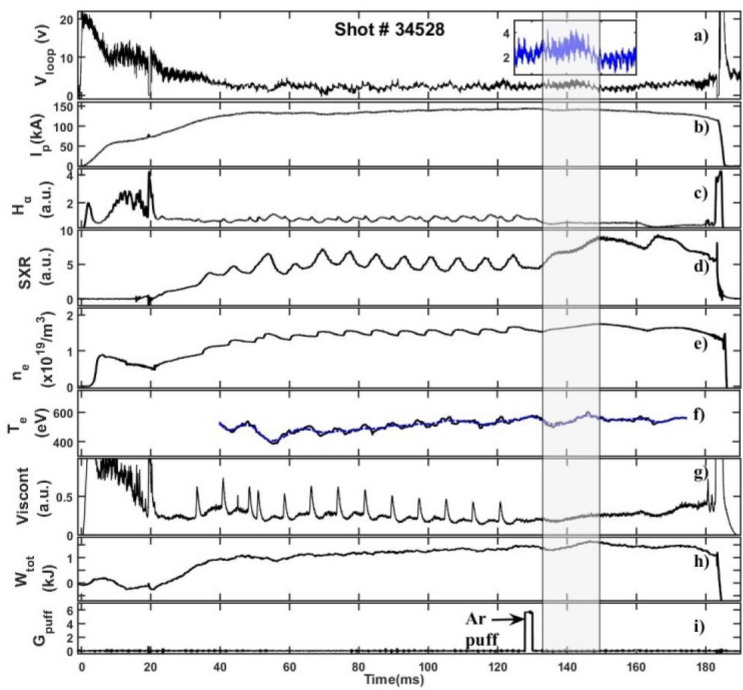
Typical ohmic discharge of the Aditya-U plasma for shot no. 34528. (**a**) Loop Voltage; (**b**) Plasma Current; (**c**) H_α_ emission intensity; (**d**) Soft X-ray emission intensity; (**e**) Electron density; (**f**) Electron temperature; (**g**) Visible continuum emission intensity; (**h**) Plasma stored energy; (**i**) Argon puff-pulse. The inset in the pannel (**a**) shows the loop voltage in the time duration of ~ 125–160 ms.

**Figure 3:**
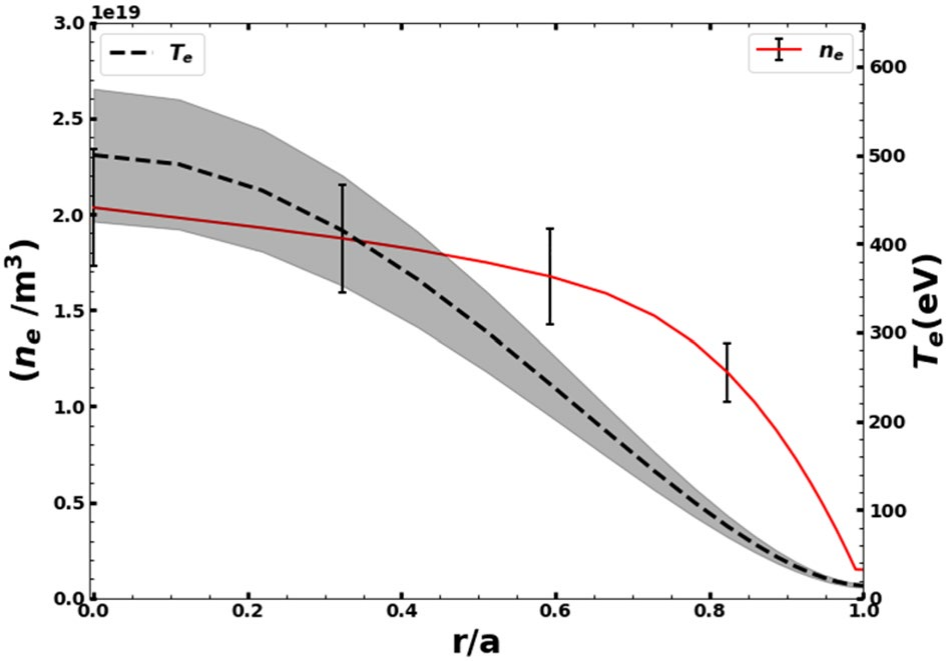
Radial profiles of n_e_ and T_e_ together with respective errorbars shown by vertical lines and shaded region for shot no. 34528.

Temporal evolution of plasma parameters of a representative discharge (shot no. 34528) of Aditya-U tokamak with an argon gas puff during the current flat-top phase is shown in Fig. 2. The Fig. 2 panels 2a) to 2i) show the temporal evolution of loop voltage, plasma current, H_α_ emission, Soft X-ray emission, chord-averaged electron density ($\overline{{n_{e} }}$), central-chord electron temperature (T_e_), visible continuum, stored energy ($W_{ \bot }$) and the voltage pulse to the peizo-valve for Ar injection respectively. The argon gas is puffed at ~ 128 ms for two milliseconds during the plasma current flat-top phase of the discharge. Note here that for maintaining the density, multiple periodic injection of hydrogen gas-puffs are used, which are turned off prior to argon injection. The observation of periodic relaxation events in plasma parameters prior to argon injection is the result of the periodic hydrogen gas puffs^48^. As seen from the Fig. 2, after argon injection, a slight increase in the electron density and hence in stored energy is observed whereas the temperature remained almost constant before and after the argon injection. A minor decrease (< 5%) is observed in the plasma current whereas the loop voltage increases by more than 50% after the argon injection before falling below to its pre-argon injection value [Fig. 2a inset]. The maximum variation is observed in the soft-X-ray emission whose intensity doubles up after the argon injection. Moreover, a sharp decrease in the Hα signal is observed after Argon injection due to the cooling of the edge plasma. The visible and VUV emissions from various radial locations are recorded immediately after the argon gas injection during a time interval of 134–154 ms as shown by the shaded region in the Fig. 2. Central chord averaged electron density ($\overline{{n_{e} }}$) ~ 2 × 10^19^ m^−3^, the edge density (ρ = r/a ~ 0.96–1) measured using Langmuir probe ~ 2–4 × 10^18^ m^−3^ and temperature of ~ 15 eV is considered for analysis in the measurement window of spectroscopic data. The radial variation of electron density and temperature as a function of normalized plasma minor radius ρ for shot no. 34528 which are used in the STRAHL code calculations are shown in Fig. 3. The radial profile of temperature is reconstructed based on the measured core temperature and edge electron temperature^49^.

**Figure 4:**
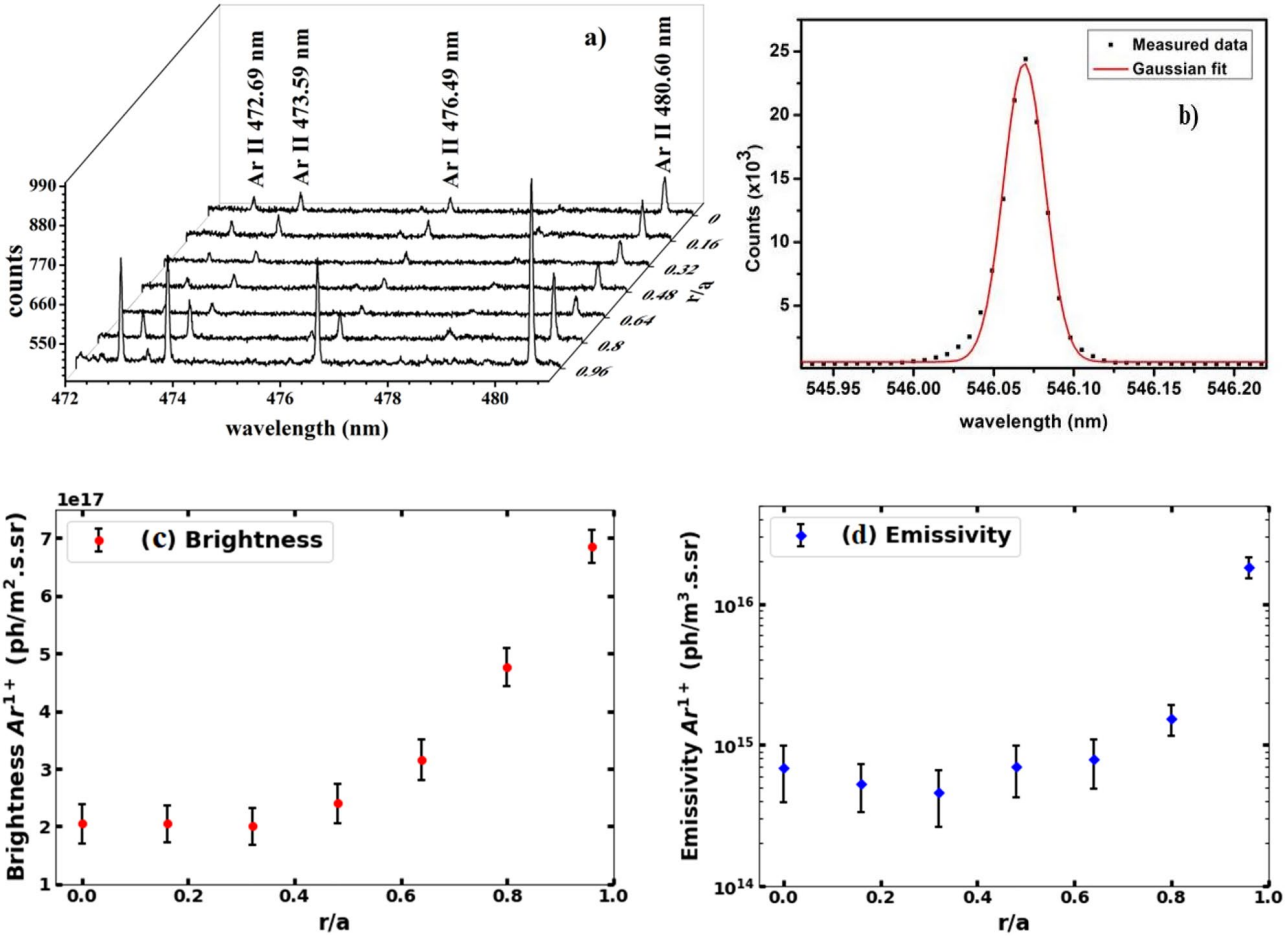
(**a**) Space resolved spectra of Ar^1+^ line emissions from seven lines of sight for shot no. 34528, (**b**) Instrumental function of the visible spectrometer obtained using Hg I line emission at 546.07 nm, (**c**) Measured brightness (red-dot) and (**d**) estimated emissivity (blue color-diamond) profile of Ar^1+^ emission with errorbars.

Figure 4a presents various Ar^1+^ spectra recorded from seven lines of sight for the discharge no. 34528 after argon injection. The entrance slit width has been kept at 100 μm and emission is collected for 20 ms after argon injection during the time interval of 134–154 ms into the discharge. The instrumental spectrum of Hg I at 546.07 nm at 100 μm slit width is shown in Fig. 4b. Experimental data points (solid squares) are fitted with Gaussian line shape (solid line). The line emissions of Ar^1+^ ions are identified using the NIST atomic spectra database^50^. The argon gas-puff results in observation of Ar^1+^ line emissions at 472.69 nm (3p^4^4s ^2^P_3/2_–3p^4^4p ^2^D_3/2_), 473.59 nm (3p^4^4s ^4^P_5/2_–3p^4^4p ^4^P_3/2_), 476.49 nm (3p^4^4s ^2^P_1/2_–3p^4^4p ^2^P_3/2_), 480.60 nm (3p^4^4s ^4^P_5/2_–3p^4^4p ^4^P_5/2_) as identified in Fig. 4a. This observation suggests that signly ionized argon remains localized in the edge region of the plasma column after argon injection. The Ar^1+^ spectra is fitted with Gaussian line-shapes and brightness of the emission is obtained by integrating the fitted Gaussian line shapes. Figure 4c shows the radial variation of the measured brightness of Ar^1+^ emission at 480.60 nm as a function of ρ (= r/a), which has been found to be peaking near plasma column boundary at r ~ 24 cm and decreases towards the interior of the plasma column. All other Ar^1+^ emissions also show similar radial variations.

The radial emissivity profile is obtained from the chord integrated brightness measurements of Ar^1+^ line emission by applying Abel-like matrix inversion technique^39^. In this technique, the plasma volume is divided into radial zones with constant emissivity, $E_{j}$, temperature and velocity. Total coverage of a particular line of sight is sum of its radial extent in each zone. The brightness, $B_{i}$, of a line of sight *i* is given by $B_{i} = \sum L_{ij} E_{j}$ or $E_{j} = \sum L_{ji}^{ - 1} B_{i}$, where subscripts *i* and *j* denote the line of sight and emission zone respectively. $L_{ij}$ is the length matrix representing path length of *i*th line of sight through *j*th zone. The emissivity, $E_{j}$, is obtained by inverting the above equation thereby calculating inverted legnth matrix, $L_{ji}^{ - 1}$. The inversion algorithm has been tested by generating synthetic chord integrated profiles of emission. In order to test the sensitivity of the inversion algorithm, different amounts of white noise are incorporated into the simulated brightness profile^51^. As mentioned previously, brightness from the observed spectrum is calculated by fitting the Gaussian profile using a least square fitting routine. Standard errors in amplitude from a Gaussian fit are obtained assuming a normal distribution of the detector noise, which depends primarily on the S/N ratio (SNR) in the brightness observed. In order to calculate SNR, photon, dark and read out noises have been considered. Uncertainty in the fitting procedure to compute the area under the curve is ≤  ~ 2%, which essentially depends on the S/N ratio, in which the large uncertainty occurs in signal with lowest amplitude. All information necessary to carry out inversion is contained in the inverted matrix [L_ij_]^−1^. The error propagation in the inversion is determined by considering ${\upsigma }_{b}^{2}$ = [${\text{L}}_{ij}^{2}$] ${\upsigma }_{a}^{2}$, where ${\upsigma }_{b}$ and ${\upsigma }_{a}$ are the standard deviations of vectors b and a; b and a represent brightness and emissivity vectors, respectively, operated by the matrix [$L_{ij}$]; b = [$L_{ij}$]a. The matrix [L_ij_] is solely based on the geometry and hence the error in [L_ij_] is negligible. Note that only the intensity of Ar^1+^ emission is inverted and inversion of line-width is not considered in the present study.

The radial variation of emissivity of Ar^1+^ line emission at 480.60 nm, obtained by inverting the measured brightness profile is shown in Fig. 4d. The errorbars in both brightness and emissivity are calculated using uncertanities mentioned earlier and shown in Fig. 4c and d respectively. The error in brightness is ~ 5–20% in all the chords with inner chords having more error. Correspondingly, the error in the emissivity profile is in the range of ~ 20–40%. As seen in Fig. 4(d), Ar^1+^ emissivity peaks at the plasma boundary, ρ ~ 0.96 and decreases significantly from ρ = 0.8 towards the core of the plasma. This emissivity profile of Ar^1+^ line emission is modelled using impurity transport code STRAHL, along with the VUV measurements to estimate the transport parameters in the edge as discussed in the next section.

**Figure 5:**
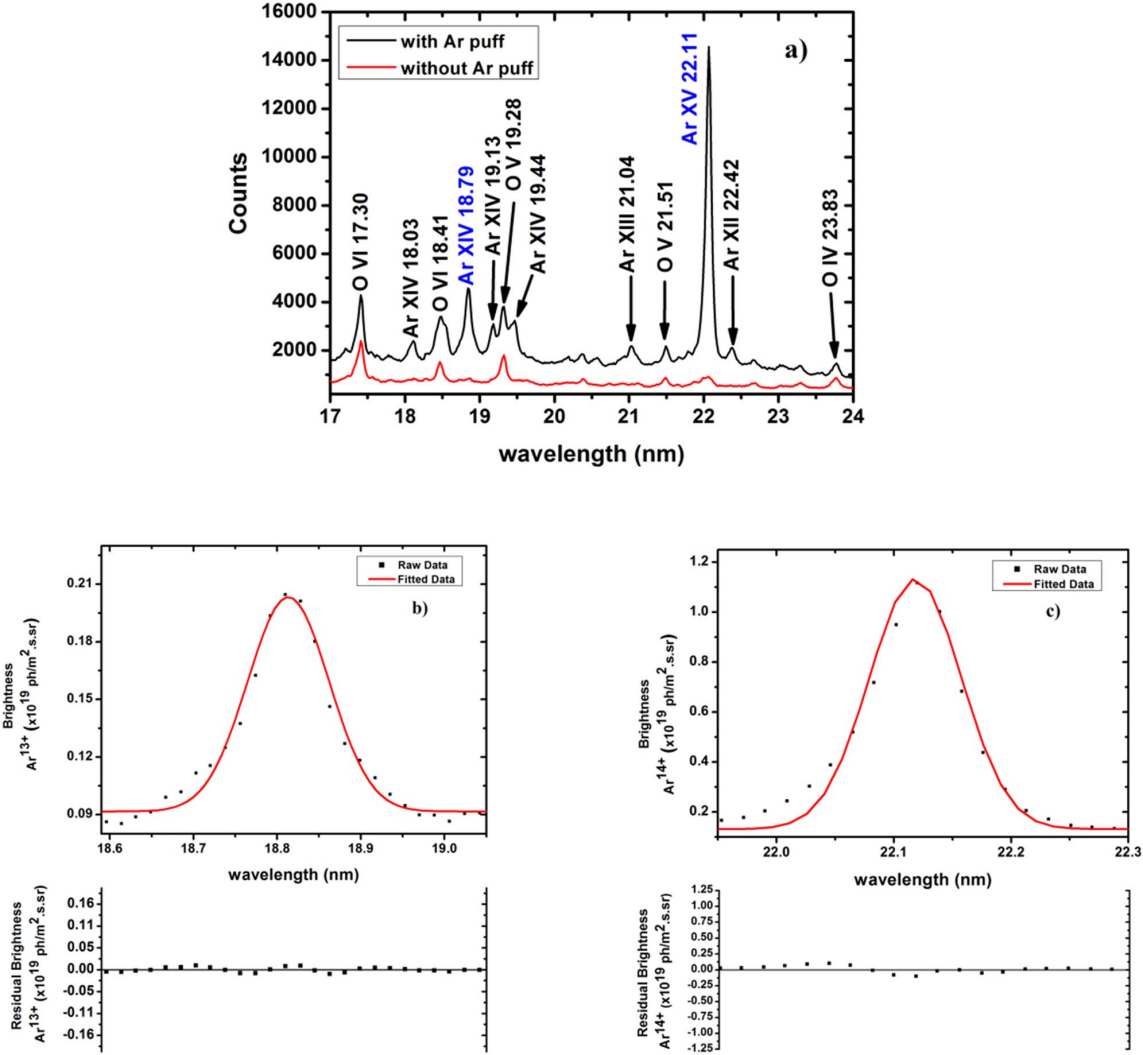
(**a**) VUV spectrum in the wavelength range of 17–24 nm recorded without (red curve) and with (black curve) argon puff for shot no. 34528 and experimental brightness of Ar^13+^ (**b**) and Ar^14+^ (**c**) line emissions at 18.79 and 22.11 nm respectrively for shot no. 34528 showing Gaussian fittings together with residues.

Figure 5a shows VUV spectral emissions captured in the wavelength range of 17–24 nm with and without argon gas puff. The black curve shows the spectrum from shot no. 34528 during the current flat top after the argon injection at ~ 128 ms into the discharge, whereas, the VUV spectra captured without argon gas puff is shown by the red curve. The exposure time of ~ 12 ms is optimised to capture multiple scans before and after the argon gas puff with sufficient signal strength. It can be seen from Fig. 5a that, in the absence of active argon injection, the VUV spectra mainly contain multiple line emissions from oxygen impurity; which is an intrinsic impurity for Aditya-U plasma. However, the VUV spectra captured immediately after the argon gas-puff contains spectral emissions from both argon and oxygen .

The emission spectral lines are identified using the NIST atomic spectra database^50^ and reconfirmed with previous studies in other tokamaks^30,52,53^. As seen from the figure, the Ar^14+^ line emission at 22.11 nm (2s^2^ ^1^S_0_–2s2p ^1^P_1_) has maximum intensity. The other spectral line which has relatively higher intensity is identified as Ar^13+^ emission at 18.79 nm (2s^2^2p ^2^P_3/2_–2s2p^2^ ^2^P_3/2_). The resonance transition of Ar^13+^ at ~ 18.03 nm is also observed, however its intensity is much lower than Ar^13+^ and Ar^14+^ lines at 18.79 nm and 22.15 nm respectively. Another resonance transition of Ar^13+^ at ~ 18.34 nm is found to be blended with O VI line at 18.41 nm in the spectra. The intensity of this resonance transition is also quite less than Ar^13+^ line emission at 18.79 nm as reported earlier by Biel et al.^54^. These observations are inline with the previously reported VUV spectra of argon in the wavelength range of 17–24 nm, where the resonance lines are observed to be having much less intensity than the other Ar^13+^ and Ar^14+^ lines^53^.

The intensity calibrated Ar^13+^ and Ar^14+^ spectral lines and the respective Gaussian fitting to the measured spectra is shown in Fig. 5b and c respectively. The residues of Gaussian fitting to the spectral lines are also shown below the respective spectra. The Gaussian fitting of VUV spectral line is determined by the apparatus function. As done in case of Ar^1+^ brightness calculations, the brightness of Ar^13+^ and Ar^14+^ is estimated by integrating the fitted Gaussian line shapes. The experimental ratio of brightness of Ar^13+^ ($I_{{Ar^{13 + } }}$) to the brightness of Ar^14+^ ($I_{{Ar^{14 + } }}$) is found to be, $I_{{Ar^{13 + } }} /I_{{Ar^{14 + } }}$ $\sim 0.23 \pm 15\%$. Error in the brightness remains within 15% after considering the uncertainty in the fitting procedure, the statistical error originating from shot-to-shot variations as well as in the intensity calibration of the spectrometer. The measured ratio of brightness is used along with Ar^1+^ emission measurements for the argon transport parameter as discussed in the next section.

## Results of transport analysis

It should be noted here that simultaneous measurements of visible and VUV argon line emissions complement each other in order to determine the impurity transport over the entire plasma minor radius. The Ar^1+^ line emissions dominate in the edge of the plasma where as Ar^13+^ and Ar^14+^ dominate in the core of the plasma. Furthermore, the edge space-resolved measurements of Ar^1+^ helps in accurate determination of the amount of argon reaching the plasma boundary and provides an appropriate boundary condition to the simulation code. Hence, simultaneous measurements of the emissivity profile of Ar^1+^ line emission and ratio of brightness of Ar^13+^ emission to that of Ar^14+^ emission allow for unique determination of impurity transport from core to edge of the Aditya-U plasma.

The atomic data in the STRAHL transport code is fetched from the ADAS database^34^, which considers the Collisional Radiative Model (CRM) for the atomic data. The line intensities of both the visible and VUV emissions have contributions from excitation, recombination, inner-shell ionization and the charge-exchange. Although, during the steady-state phase of operation, plasma is in the ionizing stage and there is no neutral beam heating, the contribution of radiative and charge-exchange recombination^55^ in the ionization balance of Ar^1+^ may not be ignored. The combined time scale of the radiative and charge exchange recombination processes is close to the measurement time-scales (~ 20 ms). Furthermore, as the upper levels are directly populated by the charge exchange process, the effect of charge exchange may not be neglected in studied x-ray spectra^56^. However, due to the limited availability of the cross-sections data of these processes for the transitions studied in this manuscript, they are not included in this study. The cross-sections of radiative and charge exchange recombination are required to be calculated and experimentally measured in future for enhancing the modelling results.

During the present study, radial emissivity profiles of Ar^1+^, Ar^13+^ and Ar^14+^ spectral emissions at 480.60 nm, 18.79 nm and 22.11 nm respectively have been calculated using the ${\upvarepsilon }^{exc}$ values for the (3p^4^4s ^4^P_5/2_–3p^4^4p ^4^P_5/2_) transition at 480.60 nm, for the (2s^2^2p ^2^P_3/2_–2s2p^2^ ^2^P_3/2_) transition at 18.79 nm and for the (2s^2^ ^1^S_0_–2s2p ^1^P_1_) transition at 22.11 nm respectively. Note here that ${\upvarepsilon }^{exc}$ values for the above transitions are not readily available in the ADAS database. In order to obtain the ${\upvarepsilon }^{exc}$ values, ionization and excitation cross-sections and rate-coefficients corresponding to Ar^1+^, Ar^13+^ and Ar^14+^ spectral transitions at 480.60 nm, 18.79 nm and 22.11 nm respectively have been obtained from ADAS database and the ${\upvarepsilon }^{exc}$ values are obtained using general collisional radiative model module available in the ADAS database.

### Determination of profiles of argon transport coefficients

**Figure 6:**
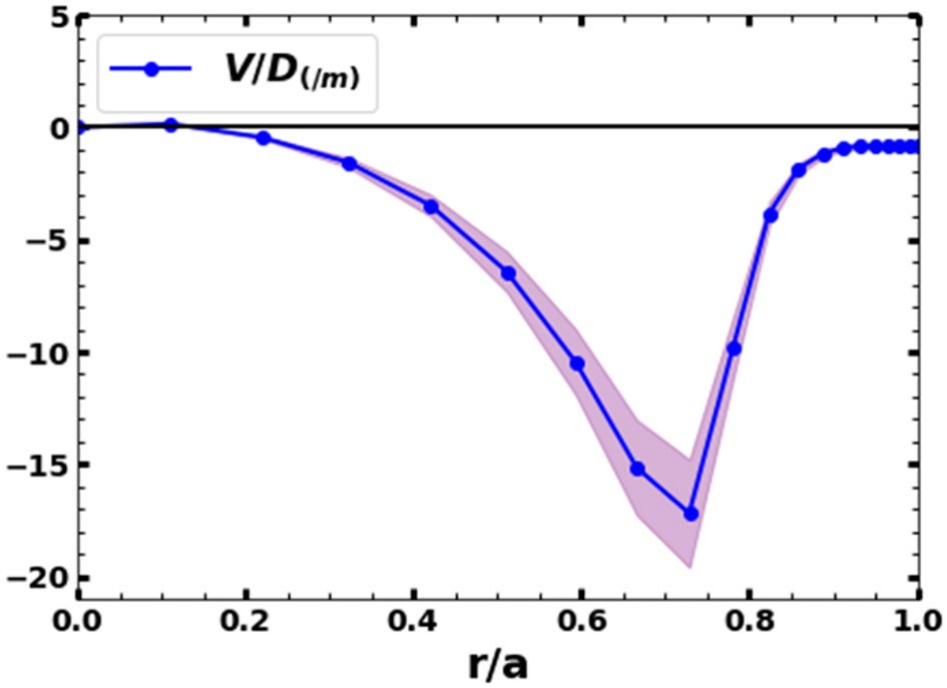
v/D ratio as a function of r/a for the best fit to the measured radial profile of Ar^1+^ and the intensity ratio of Ar^13+^ and Ar^14+^. The shaded area shows the errorbars.

The methodology described in previous section is applied to determine argon transport coefficient profiles for the shot no. 34528 after argon injection. The simulations are run by varying the convective velocity to the diffusivity ratio to match the measured Ar^1+^ emissivity profile and the intensity ratio of Ar^13+^ and Ar^14+^. A negligibly small constant value of the convective velocity is used during the initial runs, as reported in a few previous studies^54,57^, and the diffusivity is varied to match the measured Ar^1+^ emissivity profile and the intensity ratio of Ar^13+^ and Ar^14+^. The impurity source rate is also varied. Both the Ar^1+^ emissivity and its radial location is found to be sensitive to the radial profile of diffusivity and the impurity source rate. However, the brightness ratio of Ar^13+^ and Ar^14+^ line emissions does not match with the experimentally measured value for a wide range of diffusivity values and for the impurity source rate range of injected argon particles. A good match between the simulated brightness ratio of Ar^13+^ and Ar^14+^ line emissions together with the Ar^1+^ emissivity profile with the experimental measurements is only achieved when a finite value of convective velocity is introduced in the simulation. The best match is obtained for a very narrow range of valued of v/D, which is shown in Fig. 6. The error in v/D, shown by the spread in Fig. 6, remains within ~ 15% considering the errors due to white noise error in spectral measurements, fitting of the spectra and the uncertainties in the plasma density and temperature measurements. As seen from Fig. 6, the ratio of convective velocity to the diffusivity, v/D, peaks around at ρ ~ 0.75 and its maximum value is − 17 m^−1^. Similar radial profiles of convective velocities have been reported for He and Ne transports in the ASDEX Upgrade tokamak^58^. It should be noted that, during these runs, the NEOART module of the STRAHL code, which calculates the classical and neoclassical transport coefficients, has been kept off.

**Figure 7:**
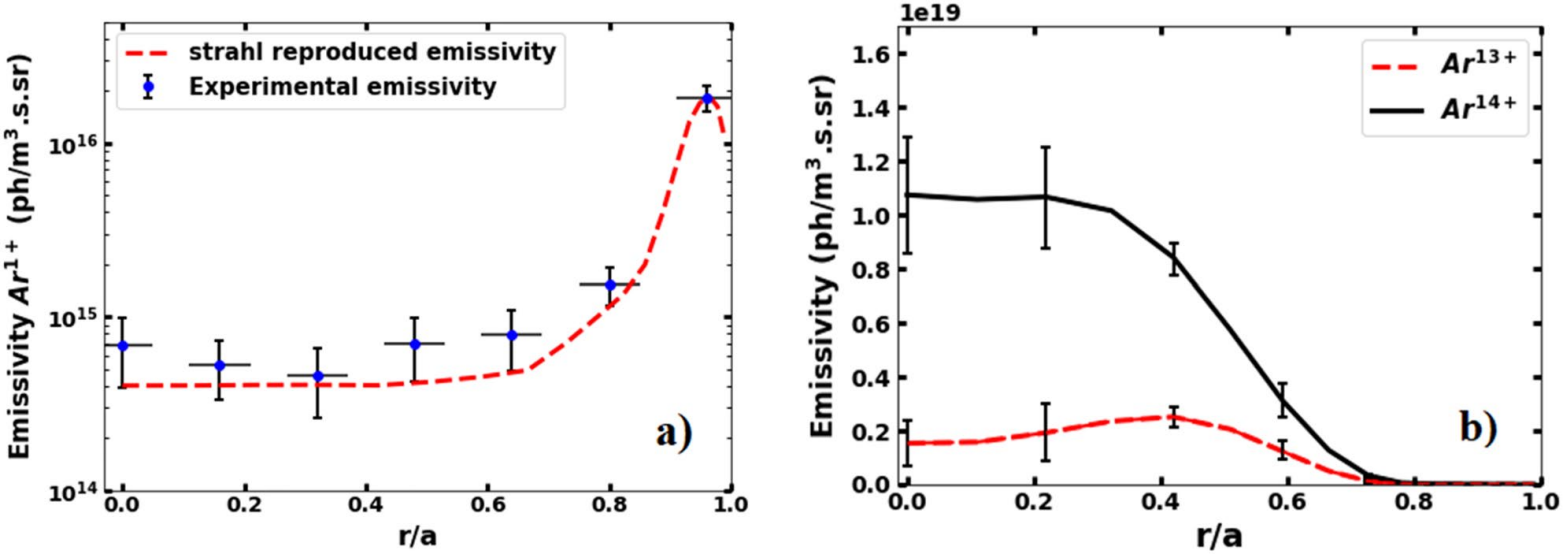
Radial emissivity profiles of (**a**) Ar^1+^ line emission (Experimental- blue dotted with errorbars) and STRAHL simulated (red dashed) and (**b**) Ar^13+^ and Ar^14+^ line emissions with errorbrs.

Figure 7a shows the best match between the experimentally measured radial emissivity profile of Ar^1+^ line emission at 480.60 nm for the discharge no. 34528 and that simulated using the STRAHL code using the radial profile of v/D shown in Fig. 6. As one can expect for the typical edge temperature range of the Aditya-U plasma of about ~ 12–15 eV, Fig. 7a shows that Ar^1+^ line emission dominates in the edge of the plasma. Note here that an argon source rate of ~ 6 × 10^18^ particles/sec is required to match the measured Ar^1+^ radial profile. The simulated radial emissivity profiles of Ar^13+^ and Ar^14+^ required for matching the measured ratio of chord-averaged intensities of these two spectral emissions is shown in Fig. 7b. Note that the simulated radial emissivity profiles of Ar^13+^ and Ar^14+^ obtained using the STRAHL code using the radial profiles of density and temperature, have been line integrated to obtain the simulated brightness of each emission before their ratio is compared with the experimental values. Furthermore, a sensitivity analysis of the obtained radial distribution of Ar^13+^ and Ar^14+^ from STRAHL has been carried out by considering the error bars present in the radial profiles of temperature (Fig. 3) for matching the measured intensity ratio. The results are folded in the errors in the radial distribution of Ar^13+^ and Ar^14+^ emissivities obtained from STRAHL and shown by the errorbars in Fig. 7b. Both the emissions have been observed to be peaking in the core region of the plasma with Ar^13+^ emission being maximum at ρ ~ 0.4, whereas Ar^14+^ emission remains almost constant at its peak value between ρ ~ 0 and 0.3.

**Figure 8:**
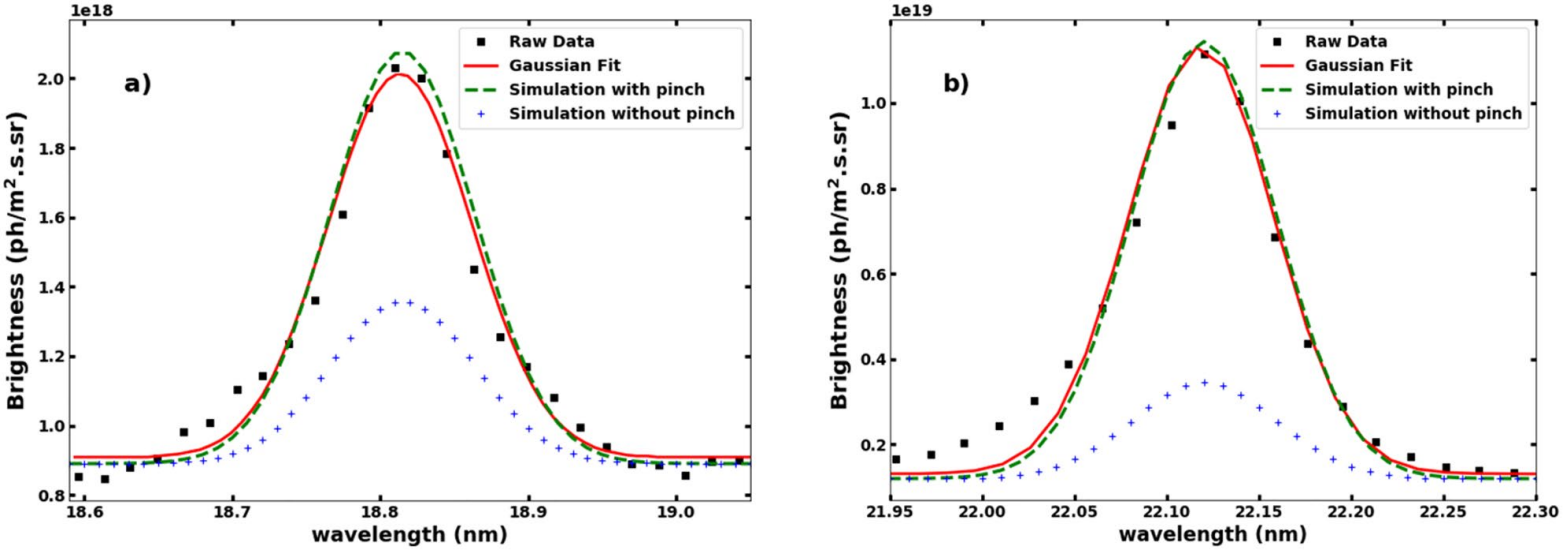
Comparison of the experimental and STRAHL simulated brightness profiles of Ar^13+^ (**a**) and Ar^14+^ (**b**) line emissions at 18.79 and 22.11 nm respectrively showing raw data (black sqaure), Gaussian fitted data (red line), STRAHL simulated brightness with (green dash) and without (blue plus sign) the effect of pinch for shot no. 34528.

The matching of the brightness ratio of Ar^13+^ and Ar^14+^ by varying the v/D ratio using STRAHL is also constrained by the matching of the individual intensities of measured Ar^13+^ and Ar^14+^ spectra folded with the instrumental function of the VUV spectrometer. Using the radial profile of emissivities obtained from STRAHL (Fig. 7b) for the v/D ration of Fig. 6 and the instrumental function of the spectrometer, the brightness spectra of Ar^13+^ and Ar^14+^ are generated. The constructed brightness spectra are superimposed to the measured spectra and shown in Fig. 8. The ratio of intensities of Ar^13+^ and Ar^14+^ obtained from STRAHL can also be matched with the measured ratio within 15% when the convective velocity is considered neglible in the iterations. However, as plotted in Fig. 8 by blue line (plus symbol), the individual line intensities of Ar^13+^ and Ar^14+^ does not match with the measured intensities without considering a finite value of convection.

**Figure 9:**
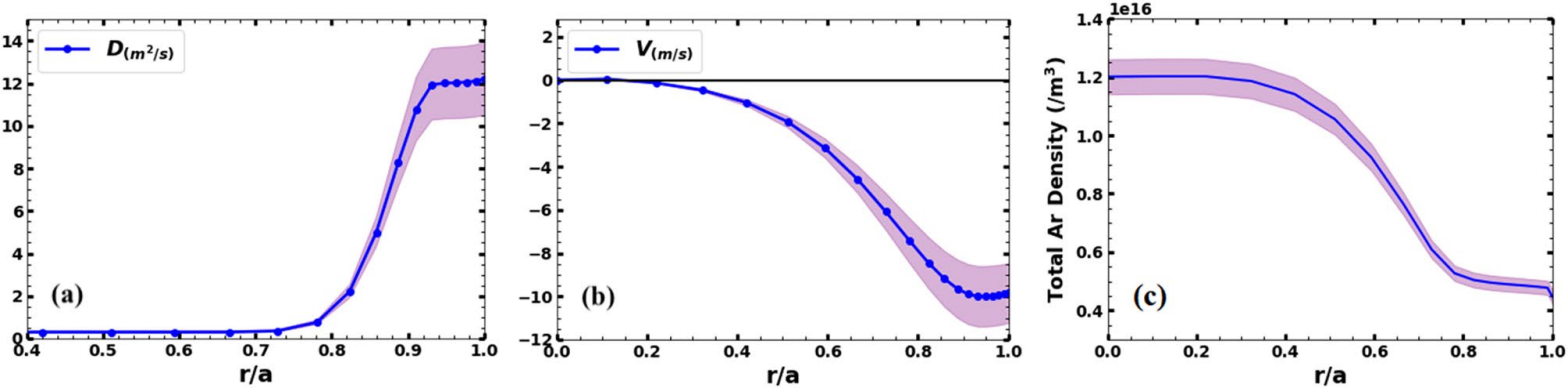
Radial profiles of Ar (**a**) diffusivity, (**b**) convective velocity and (**c**) total argon density. The shaded area shows the errorbars.

To understand the physical mechanisms driving the transport coefficients, the radial profiles of the v and D are separately plotted in Fig. 9a and b corresponding to the radial profile of v/D ratio obtained from STRAHL for the best fit of measured spectra (Fig. 6). As mentioned earlier, the measured brightness ratio of Ar^13+^ and Ar^14+^ line matches in a very narrow range of v/D ratio. Mean values of these profiles are shown with blue dot line and shaded area denotes the errorbars corresponding to the errors in the v/D ration (Fig. 6). The radial profile of argon density obtained using the diffusivity and convective velocity profiles is shown in Fig. 9c.

## Discussion

In these experiments, the spectroscopic measurements are carried out after whole of the argon-puff-pulse is over, i.e., the transient argon injection phase is not present in the measurements. Incorporating the measured radial emissivity profile of Ar^1+^ and the ratio of chord-averaged intensities of Ar^13+^ and Ar^14+^ in the STRAHL code, the diffusivity and convective velocity of argon is estimated for the Aditya-U discharges. The simultaneous measurement of Ar^1+^ provides the actual number of argon entering into the plasma. Furthermore, solving for the Ar^1+^ density using a single diffusion convection equation is a reasonable approximation considering an immediate thermalisation to the background plasma temperature. As shown in Fig. 3, due to the injection of ~ 10^17^ argon particles, the perturbation in electron density, plasma stored energy and plasma current is ~ 10%^9,10^, the perturbation in electron temperature is negligible. Hence, steady-state condition of the background plasma after the argon injection is justified. After the argon injection, the soft-X-ray emission increases significantly by a factor of 2 and the line-averaged H_α_ emission intensity decreases. Interestingly, the loop voltage increases initially after the argon injection by ~ 50% and then deceases even below to its pre-argon injection values.

As mentioned in the previous section, the radial profiles of diffusivity and convective velocity of argon are estimated from the STRAHL code using the visible and VUV measurements of radial emissivity profiles of Ar^1+^ and the ratio of intensities of Ar^13+^ and Ar^14+^. The radial profiles of diffusivity and convective velocity is shown in Fig. 9a and b respectively. The obtained radial profiles of diffusivity and convective velocity of argon show that in the core region of plasma, the argon diffuses with ~ 0.3 m^2^/s with a convective velocity in the range of ~– 0.2 to – 2 m/s. The diffusivity is found to be almost constant through the mantle and core region of the plasma. The diffusivity increases sharply beyond ρ ~ 0.8 and attains a maximum value of ~ 12 m^2^/s at ρ ~ 0.96. The convective velocity increases gradually from the core and maximizes to a value of ~– 10 m/s at ρ ~ 0.96. Both the diffusivity and the convective velocity are found to peak in the edge region. The sharp increase in the diffusivity profile beyond ρ ~ 0.8 is concomitant with the experimentally observed electron density gradient from ρ ~ 0.8 shown in Fig. 3. It has been observed that diffusivity plays dominant role in the edge compared to convection in order to match the emissivity profile of Ar^1+^ emission, whereas, the brightness ratio ($I_{{Ar^{13 + } }} /I_{{Ar^{14 + } }}$) is more sensitive to the radial profile of the convective velocity and the core diffusivity values. A strong inward convective velocity, v ~ – 10 m/s (inward) at the plasma edge, as shown in Fig. 9b, is required to match the measured brightness ratio. Furthermore, the total argon radial density profile (Fig. 9c) shows a broad peak in the central region, sharper than the density peak. This indicates concentration of argon in the plasma core region consistent with the soft X-ray emission increase, as can be seen from Fig. 1d. Note here that the minimal density increase after the argon injection does not account for the increase in the observed SXR emission. Limited by the time resolution of spectral measurements, the temporal evolution of soft X-ray emission cannot be simulated, However, qualitative estimation of SXR emission intensity before and after argon injection shows the total increases in the SXR emission intensity by a factor of 2 after argon injection can be accounted for due to the accumulation of argon in the core region.

**Figure 10:**
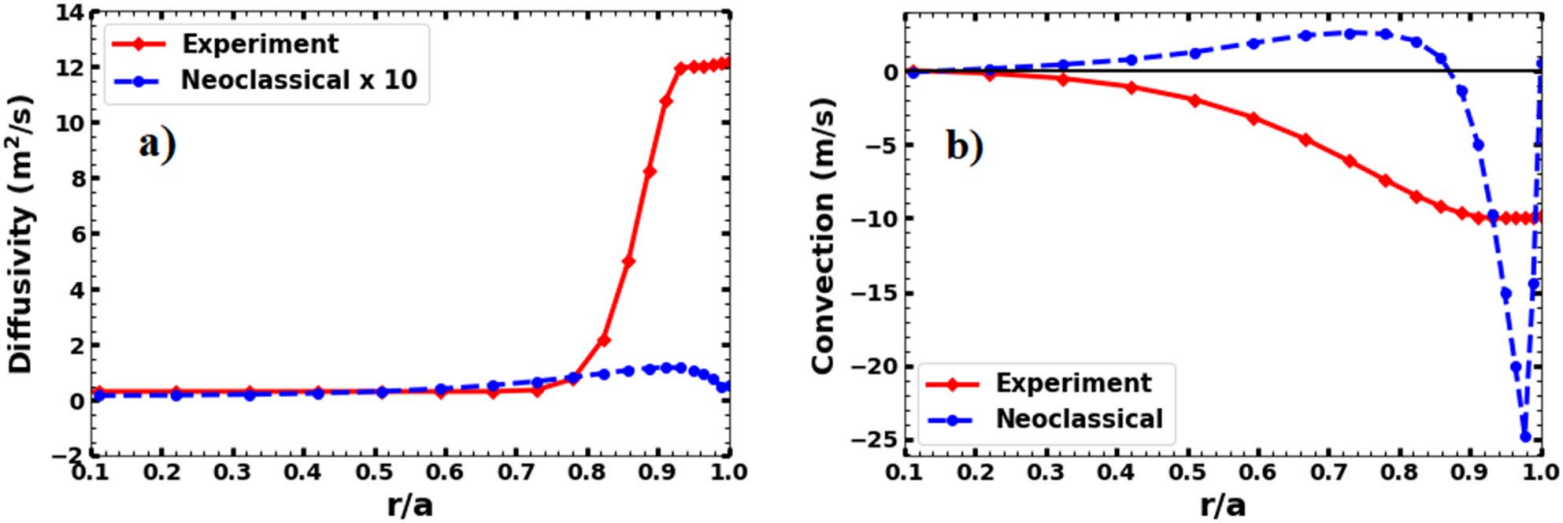
Radial profiles of argon diffusivity (**a**) and convective velocity (**b**) showing comparison between experimental (red colour diamond line) and neoclassical (blue colour dash-dot line) values. Neoclassical diffusivity has been multiplied by a factor of 10 for easy comparison with experimental values.

In order to understand argon transport mechanism in the Aditya-U plasmas, the neoclassical transport coefficients for argon has been simulated separately by switching on the NEOART module of STRAHL code using the plasma parameters of shot no. 34528. Plasma rotation has not been included in the calculation. The radial profiles of simulated neoclassical diffusivity and convective velocity using the NEOART module are plotted in Fig. 10a and b respectively for a direct comparison with those estimated from the spectroscopic measurements. It has been observed that the estimated diffusivity exceeds the respective neoclassical values in the core by an order and by more than two orders in the edge region. The radial profile of neoclassical convective velocity shows an inward convection up to ρ ~ 0.85 while the convection remains mostly outward in the mantle region. However, the estimated radial profile of the convective velocity shows that convection remains always inward with its value decreasing gradually towards the core. With only neo-classical transport, a hollow argon density profile has been observed. The total argon density peaks at ρ ~ 0.85 and does not seem to accumulate in the core. The result is found to be consistent with the fact that neoclassical convection is outwardly directed from ρ ~ 0 to 0.85 and strongly inward between ρ ~ 0.85 to 1.

The Fig. 10a and b clearly show that the argon transport in Aditya-U does not follow neoclassical behaviour in both edge and core regions. In fact, diffusivity of several impurity species including argon has mainly been observed to be anomalous in the edge region whereas it has been reported to be either neoclassical^59,60^ or anomalous^9,61,62^ in the core region of the tokamak plasmas. The turbulent driven transport remains to be one of the main reasons for the observed deviations from the neoclassical behaviour of impurity transport^61,62^. In recent years, considerable efforts have been made to understand the observed relatively higher values of impurity diffusivities and strong inward pinch, especially in the edge region. It is quite well known that different kinds of instabilities are sustained in a tokamak plasmas leading to the growth of turbulent fluctuations, which in-turn affect the transport of heat and particles. Density and floating potential fluctuations are routinely observed in the edge and SOL region in typical ohmic plasma of Aditya-U tokamak^63^. In the reported discharges of Aditya-U, the ion temperature gradient (ITG) mode driven turbulence is likely to be dominaning in the edge plasma region, given by^64,65^, $$D_{ITG} \sim \frac{{c_{s} \rho_{s}^{2} }}{{\left( {L_{p} R} \right)^{1/2} }}\left( {\frac{{q^{4} R}}{{L_{p} }}} \right)^{1/4}$$, where $$c_{s}$$: ion sound speed, $$\rho_{s}$$: ion Larmor radius, $$R$$: major radius $$q$$: edge safety factor, $$Lp$$: pressure scale length. Considering the argon ions, the values used in the calculations are $$c_{s}$$ ~ 8.66 × 10^3^ m/s, $$\rho_{s}$$ ~ 3.83 mm, R = 0.75 m, $$q$$ ~ 3.45, $$Lp$$ ~ 0.011 m^66^. The diffusivity thus calculated in the edge comes out to be ~ 13 m^2^/s; which matches quite well with the experimental edge diffusivity of ~ 12 m^2^/s. Similar results of ITG driven high diffusivity in the edge are observed in previous experiments of Aditya Tokamak for oxygen and iron impurities^47,67^. Earlier studies have also shown that the argon diffusivity decreases with an increase in the mean density following an Alcator-like scaling with mean density $$D \propto {\raise0.7ex\hbox{$1$} \!\mathord{\left/ {\vphantom {1 {\overline{{n_{e} }} }}}\right.\kern-0pt} \!\lower0.7ex\hbox{${\overline{{n_{e} }} }$}}$$^54^. The density dependency of diffusion is under investigation in Aditya-U and will be addressed in a separate communication.

As mentioned earlier, a finite value of convection velocity is necessary to match the measured radial emissivity profile of Ar^1+^ and the ratio of Ar^13+^ and Ar^14+^ emission intensities. However, again, as seen from Fig. 10b, the radial profile of simulated neoclassical value of convective velocity using NEOART module does not match with the estimated one using the spectroscopic measurements. Although, NEOART-simulated convection at the edge (up to ρ ~ 0.85) agrees in sign with the estimated one, indicating an inward motion, they differ significantly in the absolute value. Furthermore, the NEOART-simulated convective velocity changes sign, showing an outward convection, opposite to that estimated from the measurements. The estimated convective velocity always remain in inward direction throughout the plasma cross-section. These observations again suggest that neoclassical transport may not be accountable for experimental observation of argon impurity transport in the Aditya-U. Strong local inward pinch of the impurities are commonly observed in several tokamaks with different discharge scenarios such as ohmic, L- mode and H-mode^9,11,58^. With the finite loop-voltage $\left( {V = E_{\phi } \times 2\pi R,\; where\;R\;is\;the\;major\;radius} \right)$ in the reported discharges, a radial pinch due to $E_{\phi } \times B_{\theta }$, where $B_{\theta }$ is the poloidal magnetic field, always exists^48^. As mentioned earlier and can be seen from Fig. 3a (inset), loop voltage increases by 50% after argon injection, hence the pinch also increases after argon injection. After the injection of the argon gas in to the plasma through the edge region, the temperature in the edge region (ρ ∼ 0.8–1) decreases initially as the electrons lose their energy in ionizing the argon. As the temperature in the edge region decreases, the resistance of the plasma increases leading to an increase in the loop voltage^48^. The increase in loop voltage leads to inward pinch through Ware mechanism.The pinch velocity calculated using the enhanced loop voltage after argon injection comes out to be ~ 8 m/s, which is quite close to the maximum value of convective velocity obtained from the STRAHL simulation using the spectroscopic data. Hence, $E_{\phi } \times B_{\theta }$ driven pinch may be a possible mechanism of pinching the argon leading to its concentration in the plasma core.

Based on the Weiland multifliud model, curvature pinch also drives the impurity inward. Further, in case of ITG instability, pinch due to parallel impurity compression is also known to drive the impurity inward^68^. Thus, with the ITG mode driven turbulence likely to be dominating in the edge region, pinch related to parallel impurity compression may also lead to inward convection as observed in the experiments. However, in the case of discharges analysed during this study, thermo-diffusion, curvature pinch and pinch from parallel compression are negligible as argon injection does not vary the q_edge_ and magnetic shear significantly. Thermo-diffusion is found to originate from the compression of the diamagnetic drift velocity and its magnitude is inversely proportional to the charge number. As a result the thermo-diffusion pinch becomes negligible for high Z impurity^69^. Curvature Pinch is proportional to the magnetic shear and is inward for a monotonically increasing q-profile and outward for reversed q-profile. Since no significant change in plasma parameters was observed after argon injection, no change in the magnetic shear is expected after Ar injection. Lastly, pinch from parallel compression is connected with the parallel dynamics of the impurities and is proportional to $\sim 1/2q^{2}$. Since, no significant change in plasma current was observed after argon injection, no change in the q value is expected after Ar injection.

## Conclusion

Argon impurity transport in the Aditya-U ohmic plasma has been studied using a space resolved visible spectrscopic system and a VUV spectrometer. For this purpose, we injected a trace amount of argon gas into the plasma edge during its plasma current flat–top phase. The radial profile of Ar^1+^ (Cl-like) at 472.69 nm, 473.59 nm, 476.49 nm, 480.60 nm line emissions and chord-averaged VUV spectral line emissions from Ar^13+^ (B-like) at 18.79 nm and Ar^14+^ (Be-like) at 22.11 nm are measured simultaneously after the argon injection. In case of typical plasma parameters of the Aditya-U, Ar^1+^ line emission has been observed to be dominating in the edge region while Ar^13+^ and Ar^14+^ line emissions arise from the core region due to the Aditya-U tokamak’s core plasma electron temperature of ~ 500 eV. Thus, simultaneous measurements of these argon charge states provide effective constrains for the STRAHL simulation for estimating argon transport coefficient across the entire plasma minor radius. From the line integrated radial profile of Ar^1+^ line emission, its radial emissivity profile has been obtained using Abel like matrix inversion. Also, the line intensity ratio of Ar^13+^ and Ar^14+^ has been obtained from experimental measurements. In order to estimate the argon transport coefficients, both radial emissivity profile of Ar^1+^ and line ratio of Ar^13+^ and Ar^14+^ emissions have been simulated using the STRAHL code and matched with the measurements. In case of typical ohmic discharge of the Aditya-U, it has been observed that along with the diffusivity, a finite value of convective velocity is compulsorily required to match the spectroscopic measurements. The argon diffusivity in the core and mantle region is estimated to be ~ 0.3 m^2^/s, which increases sharply beyond ρ ~ 0.8, up to a maximum of ~ 12 m^2^/s at ρ ~ 0.96. Also, the convective velocity is found to be directed towards the core all over the plasma minor radius, attaining a maximum value of ~ 10 m/s at ρ ~ 0.96 decreasing gradually to ~ 0.2 m/s in the core. The diffusivity estimated using spectroscopic measurements is found to be much higher than the neoclassical values obtained using the NEOART module of STRAHL code, at all radial locations. The estimated convective velocity also show a significant deviation from the neoclassical values over the plasma radius. The ${\varvec{E}}_{\phi } \times {\varvec{B}}_{{\varvec{\theta}}}$ may be resulting in an inward convection of argon impurities leading to an argon impurity accumulation in the core of the plasma as reflected by the observation of peaked- radial profile of argon density. It is found that the argon impurity tends to accumate into the plasma core in these discharges and its transport in Aditya-U is anomalous in nature rather than neoclassical. Furthermore, the ion temperature gradient (ITG) mode driven turbulence transport may be a reason for the observation of high diffusivities of argon, particularly in the edge plasma region.

## Data Availability

The data that support the findings of this study are available from the corresponding authors upon reasonable request.
