# Author Correction: Role of pinch in Argon impurity transport in ohmic discharges of Aditya-U Tokamak

**DOI:** 10.1038/s41598-023-47362-8

**Published:** 2023-11-15

**Authors:** K. Shah, J. Ghosh, S. Patel, M. B. Chowdhuri, K. A. Jadeja, G. Shukla, T. Macwan, A. Kumar, S. Dolui, K. Singh, R. L. Tanna, K. M. Patel, R. Dey, R. Manchanda, N. Ramaiya, R. Kumar, S. Aich, N. Yadava, S. Purohit, M. K. Gupta, U. C. Nagora, S. K. Pathak, P. K. Atrey, K. B. K. Mayya

**Affiliations:** 1 Department of Physics, Pandit Deendayal Energy University, Raisan, Gandhinagar, 382 007 India; 2 Institute for Plasma Research, Bhat, Gandhinagar, 382 428 India; 3 Homi Bhabha National Institute, Training School Complex, Anushaktinagar, Mumbai, 400 094 India; 4 Department of Nano Science and Advanced Materials, Saurashtra University, Rajkot, 360 005 India; 5 ITER-India, Institute for Plasma Research, Koteshwar, Ahmedabad, 380 005 India; 6 University of California, Los Angeles, CA 90095 USA; 7 Institute of Science, Nirma University, Ahmedabad, 382 481 India

Correction to: *Scientific Reports* 10.1038/s41598-023-42746-2, published online 26 September 2023

The original PDF version of this Article contained an error in Figure 9, where the Radial profiles were incorrectly displayed.

The original PDF version of the Article has been corrected. The HTML version was correct at the time of publication.

